# Nerve influence on the metabolism of type I and type II diabetic corneal stroma: an in vitro study

**DOI:** 10.1038/s41598-021-93164-1

**Published:** 2021-07-01

**Authors:** Amy E. Whelchel, Sarah E. Nicholas, Jian-Xing Ma, Dimitrios Karamichos

**Affiliations:** 1 Department of Physiology, University of Oklahoma Health Sciences Center, 940 Stanton L. Young, Oklahoma City, OK USA; 2 North Texas Eye Research Institute, University of North Texas Health Science Center, 3500 Camp Bowie Blvd, Fort Worth, TX 76107 USA; 3 Department of Pharmaceutical Sciences, University of North Texas Health Science Center, 3500 Camp Bowie Blvd, Fort Worth, TX 76107 USA; 4 Harold Hamm Oklahoma Diabetes Center, 1000 N Lincoln Blvd, Oklahoma City, OK USA; 5 Department of Pharmacology and Neuroscience, University of North Texas Health Science Center, 3500 Camp Bowie Blvd, Fort Worth, TX 76107 USA

**Keywords:** Corneal diseases, Mechanisms of disease

## Abstract

Corneal innervation plays a major role in the pathobiology of diabetic corneal disease. However, innervation impact has mainly been investigated in the context of diabetic epitheliopathy and wound healing. Further studies are warranted in the corneal stroma-nerve interactions. This study unravels the nerve influence on corneal stroma metabolism. Corneal stromal cells were isolated from healthy (HCFs) and diabetes mellitus (Type1DM and Type2 DM) donors. Cells were cultured on polycarbonate membranes, stimulated by stable Vitamin C, and stroma-only and stroma-nerve co-cultures were investigated for metabolic alterations. Innervated compared to stroma-only constructs exhibited significant alterations in pyrimidine, glycerol phosphate shuttle, electron transport chain and glycolysis. The most highly altered metabolites between healthy and T1DMs innervated were phosphatidylethanolamine biosynthesis, and pyrimidine, methionine, aspartate metabolism. Healthy and T2DMs main pathways included aspartate, glycerol phosphate shuttle, electron transport chain, and gluconeogenesis. The metabolic impact on T1DMs and T2DMs was pyrimidine, purine, aspartate, and methionine. Interestingly, the glucose-6-phosphate and oxaloacetate was higher in T2DMs compared to T1DMs. Our in vitro co-culture model allows the examination of key metabolic pathways corresponding to corneal innervation in the diabetic stroma. These novel findings can pave the way for future studies to fully understand the metabolic distinctions in the diabetic cornea.

## Introduction

Diabetes mellitus (DM) is a systemic, metabolic disease that is one of the most significant clinical burdens today, well known for its high comorbidity^1–3^. The current diabetic population is over 34 million in the United States, significantly affecting societal wellbeing both physically and financially^4^. The most prevalent subgroups are Type 1DM (T1DM) and Type 2DM (T2DM), comprising most of the diabetic population in the U.S. T1DM is characterized by low insulin production due to autoimmune destruction of pancreatic B cells, while T2DM is a cellular state of insulin resistance due to prolonged exposure to hyperglycemia. Without the activity of insulin to help signal the body to metabolize glucose properly, glucose build-up leads to an intracellular switch in energy production, resulting in an inherently dysfunctional metabolic profile^5^. Not only does glucose metabolism become dysfunctional, but many metabolic pathways, including amino acid and fatty acid metabolism are known to be associated with insulin resistance^6^. Interestingly, both T1DM and T2DM exhibit similar clinical complications, despite their cellular and molecular differences.

In the context of the human eye, DM is known to affect the cornea, a condition termed diabetic keratopathy, with patients experiencing spontaneous epithelial lesions, neuronal degeneration, reduced sensitivity, slow wound healing, hazy vision (i.e. scarring), and dry eye^7–9^. DM-related corneal complications can ultimately lead to vision loss, severely impacting quality of life for the patient. With such complications in both T1DM and T2DM patients and up to 70% of the diabetic population experiencing mild to severe diabetic keratopathy symptoms, there is a strong drive to better understand the pathophysiology of the disease^10^.

The cornea is a rather unique tissue, with complex structure and hierarchy to maintain its homeostasis. The cornea is avascular, with nutrients and regulatory molecules delivered via the tear film, aqueous humor, and/or resident cells. The corneal stroma (the largest/thickest layer of the cornea) is composed of a highly organized collagenous extracellular matrix (ECM), secreted by resident keratocytes^11^. Sensory neurons stretch centripetally through the stroma and penetrate up into the epithelial layer, providing the sensitivity necessary to protect the eye. Nerve degeneration of these sensory neurons is among one of the first-appearing manifestations of diabetic keratopathy/neuropathy, while other symptoms, such as epithelial dysfunction, lesions and corneal scarring, often present later^12,13^. The stroma is known to play a role in the corneal scarring and edema, which can become severe and threaten vision acuity^14^. The reduced innervation and sensitivity in the diabetic cornea, in combination with the scarring and abnormal ECM composition that can take place, suggests that there is crosstalk between these two resident cell types. Corneal stromal fibroblasts have been found to express mRNA for the NGF receptor; furthermore, NGF levels are increased during the wound healing process, indicating a crosstalk between the neurons may play a role in the promotion of proper ECM production by the fibroblasts^15^. In an in vivo model of tissue repair, Wang et al.^16^ found that extracellular matrix production is improved when peripheral neurons are added to a fibroblast culture^16^. Despite this evidence that neurons impact the activity of fibroblasts, their impact on one another has sparsely been investigated in the cornea. The relationship and crosstalk between the corneal nerves and the stromal layer is not well understood, both in the context of diabetic keratopathy/neuropathy and in corneal pathobiology, in general.

Our group has previously shown that the human diabetic cornea possess a markedly different metabolic profile, when compared to healthy counterparts^17^, remarkably maintaining the diabetic phenotype (i.e. memory) in vitro^14,18^. Herein, we aim to investigate how nerves contribute to, or impact, the metabolic function of the corneal stroma microenvironment and unravel their interactions utilizing our established innervated in vitro 3D co-culture model.

## Methods

### Ethical approval and informed consent

The study was performed with the Institutional Review Board (IRB) approval from the University of Oklahoma Health Sciences Center (protocol #3450). Written and informed consent was obtained prior to tissue collection, all methods were performed in accordance with federal and institutional guidelines and all human samples were de-identified prior to analysis. This research adhered to the tenets of the Declaration of Helsinki.

### Cell isolation and culture

Human corneal fibroblasts were isolated from donor corneas of healthy, T1DM and T2DM individuals. The corneas were lightly scraped on both sides, to remove endothelium and epithelium, cut into approximately 1mm^2^ pieces, and maintained in Eagle’s Minimum Essential Medium (EMEM: ATCC; Manassas, VA), 10% fetal bovine serum (FBS: Atlantic Biological’s; Lawrenceville, CA), and 1% antibiotic-antimycotic (AA: Gibco, Life Technologies; Carlsbad, Ca) at 37 °C to promote cell expansion. Upon cell expansion, corneal explants were removed, and fibroblasts were collected and further sub-cultured (EMEM, 10% FBS, 1% AA). Fibroblasts used for experiments were between passages 3–7. The SH-SY5Y neuroblastoma cell line (Sigma-Aldrich; St. Louis, MO) was sub-cultured in the same culture conditions as the fibroblasts (EMEM, 10% FBS, 1% AA), prior to utilization in co-cultures.

### 3D in vitro constructs

Non-innervated constructs were grown as previously described^14^. Fibroblasts from healthy, T1DM, and T2DM corneas were seeded onto 0.4 mm polycarbonate membranes at a density of 1 × 10^6^ cells per well (EMEM, 10% FBS, 1% AA). The following day, cells were stimulated with 0.5 mM VitC (ascorbic acid) to promote endogenous secretion of ECM. Medium was refreshed in both the top and bottom wells three times a week for 4 weeks.

### 3D in vitro innervated constructs

Stroma-nerve co-cultures were grown as previously described^18^. Cultures were maintained for 3 weeks as described above in medium supplemented with VitC in both the top and bottom wells. At the start of week 4, SH-SY5Y neuroblastoma cells were seeded into the top well directly onto the constructs at a density of 500,000 cells per well. The bottom well was maintained in VitC medium to continue to promote ECM secretion. The following day, the top well was stimulated with 10 µM retinoic acid to induce neuronal differentiation for 5 days (EMEM, 1% FBS, 1% AA, 10 µM RA), and then switched to 2 nM BDNF for 48 h in serum-free medium (EMEM, 1% AA, 2nM BDNF). At the 4-week time point, constructs were processed for metabolomics.

### Experimental groups

A total of six different conditions were analyzed. Defined groups consisted of non-innervated healthy (HCF), T1DM and T2DMs, and innervated healthy (HCF-N), T1DM (T1DM-N) and T2DMs (T2DM-N). Grouping for analysis was predetermined and conducted between (1) innervated cultures and their respective control separately (HCF vs. HCF-N, T1DM vs. T1DM-N, T2DM vs. T2DM-N), (2) between the innervated constructs of each cell type (HCF-N vs. T1DM-N vs. T2DM-N), and (3) between the innervated constructs of the diabetics (T1DM-N vs. T2DM-N).

### Metabolite extraction

Metabolites were extracted following a previously established protocol^19^. 3D constructs were washed twice with DPBS, and then incubated at − 80°C in 80% methanol for 20 min. The samples were centrifuged at 13,500*g* and further disrupted with vortexing in 80% methanol. This process was repeated three times. Supernatants were collected and lyophilized until completely dry to produce a small metabolite pellet, which were then re-suspended in HPLC-grade water. These samples were injected and analyzed using a hybrid 5500 QTRAP triple quadrupole mass spectrometer (AB/SCIEX) coupled to a Prominence UFLC system (Shimadzu) using an Amide HILIC column (Waters), armed with a selected reaction monitoring (SRM) with positive/negative polarity switching. Peak areas from the total ion current were integrated using MultiQuant v2.1 software (AB/SCIEX). Integrated peak intensities represented relative metabolite abundance.

### Statistical analysis

All metabolites were analyzed using Metaboanalyst, Excel, and GraphPad PRISM 8. Metaboanalyst was utilized for enrichment and pathway analysis. Excel analysis was utilized to determine a 2-fold differential in metabolites expression, as well as those that exhibited significance of p ≤ 0.05 among groups. Metabolites that passed the “2-fold” cut-off were analyzed on GraphPad PRISM 8 for statistical significance. Measurements were taken in triplicate, with 3–4 technical replicates per donor type. Student’s t test and ANOVA were utilized, when necessary.

## Results

### Metabolic impact of neuronal presence in healthy and diabetic constructs

**Figure 1:**
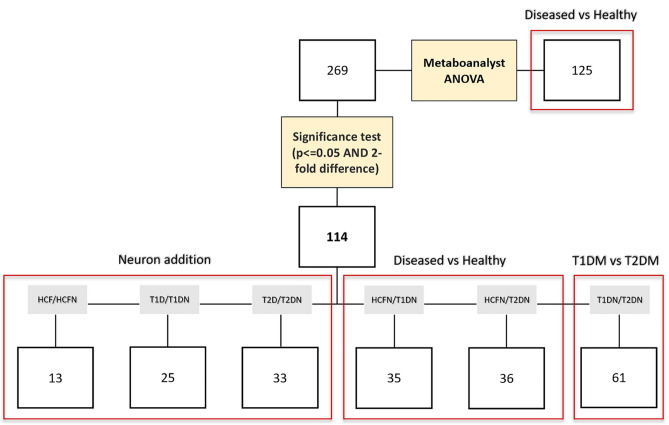
Summary of metabolic impact of neuronal presence in HCFs, T1DMs, T2DMs. A total of 269 metabolites were analyzed via one-way ANOVA on Metaboanalyst. Individual t tests were utilized for the 6 comparisons listed above: HCF/HCF-N, T1D/T1DM-N, T2D/T2DM-N, HCF-N/T1DM-N, HCF-N/T2DM-N, T1DM-N/T2DM-N. Only metabolites that reached a 2-fold change and a significance of p < 0.05 were deemed significant. All samples analyzed for metabolites had a n = 3–4.

**Figure 2:**
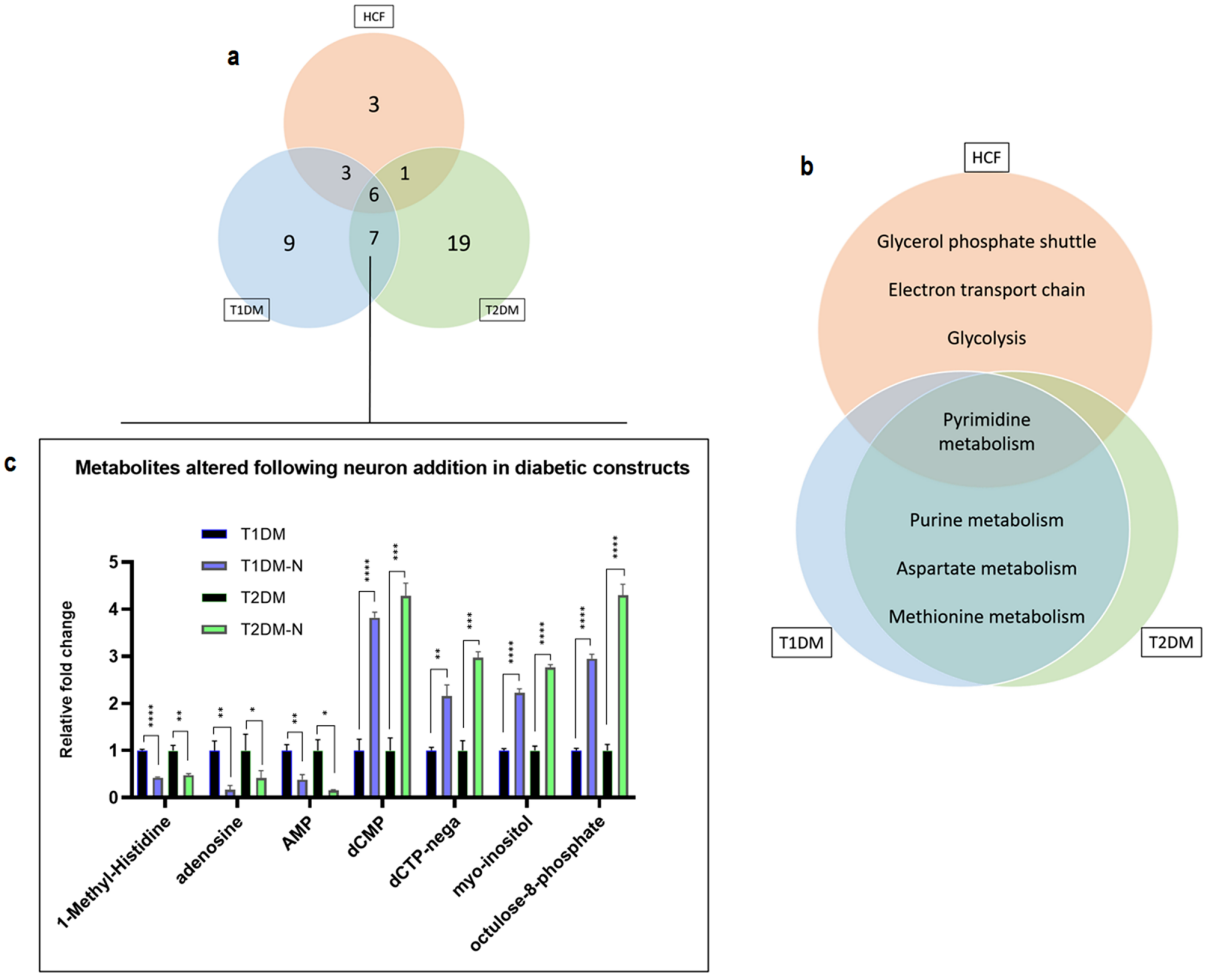
The effect of neurons on metabolism. Representative Venn diagrams of (**a**) the number of shared metabolites that were altered following neuron addition, and (**b**) the top four most significantly altered pathways for each cell type following neuron addition. (**c**) Graph exhibiting all metabolites significantly altered in both the diabetic constructs and not the healthy, following neuron addition. n = 3–4, student’s t test, p: *≤ 0.05; **≤ 0.01; ***≤ 0.001; ****≤ 0.0001.

To understand how the addition of neurons impacted the metabolic profile of the 3D constructs for each condition, three different groups were compared: (a) HCF vs. HCF-N, (b) T1DM vs. T1DM-N, and (c) T2DM vs. T2DM-N. Out of 269 metabolites analyzed, the number of metabolites that reached both a 2-fold change in concentration and a significance of p < 0.05, as a result of the introduction of neurons into the system, was 13, 25, and 33 for HCF, T1DM, and T2DM constructs, respectively (Fig. 1). 6 metabolites were either upregulated or downregulated across all three conditions, 3 were shared between the healthy and T1DM, 1 shared between the healthy and T2DM, and 7 were shared between the T1DM and T2DM (Fig. 2a). In a Metaboanalyst pathway analysis of all metabolites altered within each condition separately, the results revealed similarities between the T1DM and T2DM, as the top four most highly affected pathways were the same. The top four pathways for both T1DM and T2DM groups were pyrimidine metabolism (p = 0.00975 for T1DM, p = 0.000266 for T2DM), purine metabolism (p = 0.0247, p = 0.00024), aspartate metabolism (p = 0.0453, p = 0.0036) and methionine metabolism (p = 0.755, p = 0.00154). When healthy constructs were analyzed relative to their innervated counterparts, they also exhibited significant alterations in pyrimidine metabolism (p = 0.0344), as well as the glycerol phosphate shuttle (p = 0.00768), the electron transport chain (p = 0.0225) and glycolysis pathway (p = 0.0379) (Fig. 2b). The metabolites common between the T1DM and T2DM constructs include 1-methylhistidine, adenosine, adenosine monophosphate, deoxycytidine monophosphate, deoxycytidine triphosphate, myoinositol, and octulose 8-phosphate (Fig. 2c). A detailed list on the contributing metabolites to the determination of these pathways can be found in the supplemental data (Supplemental Table 1).

### T1DMs and T2DMs exhibit significant alterations in key energy production metabolic processes

**Figure 3:**
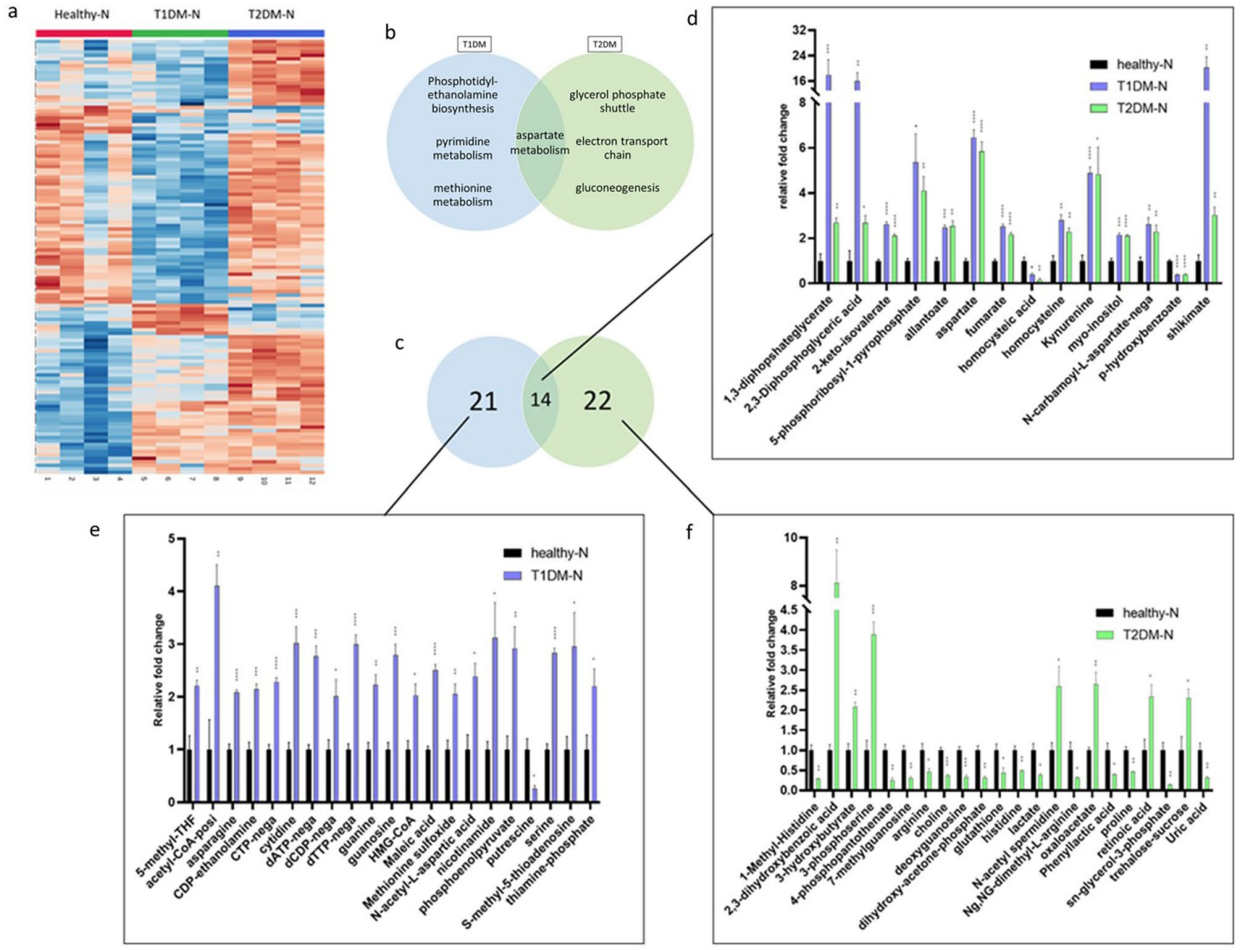
The differences between the diabetic innervated constructs from the healthy. (**A**) Heat map representing all significant metabolites found between the healthy, T1DM and T2DM innervated constructs, as determined by one-way ANOVA on Metaboanalyst. (**B**) The top 4 pathways most significantly altered between the T1DM innervated constructs and the healthy, and the T2DM innervated constructs and the healthy. (**C**) The number of metabolites altered in the T1DM and T2DM innervated constructs when compared to the healthy. (**D**–**F**) graphs exhibiting (**D**) metabolites altered in both T1DM and T2DM innervated constructs, (**E**) those of the T1DM innervated constructs, and (**F**) those of the T2DM innervated constructs. n = 3–4, student’s t test, p: *≤ 0.05; **≤ 0.01; ***≤ 0.001; ****≤ 0.0001.

Further analysis was conducted between the innervated constructs to determine key metabolic differences between the healthy and diabetics. In total, 125 metabolites were determined to be significantly altered between the healthy and diabetic innervated cultures (Fig. 3A; Supplemental Table 2). Pathway analysis (Fig. 3B) revealed that the most highly altered metabolites between the healthy and T1DM innervated constructs fell within the metabolic pathways of phosphatidylethanolamine biosynthesis (p = 0.00652), pyrimidine metabolism (p = 0.0124), methionine metabolism (p = 0.0025), and aspartate metabolism (p = 0.00538). For the healthy and T2DM constructs (Fig. 3F), the top four pathways also included aspartate metabolism (p = 0.00538), as well as glycerol phosphate shuttle (p = 0.00501), electron transport chain (p = 0.00308), and the gluconeogenesis pathway (p = 0.00538). A total of 14 metabolites that reached significance were shared between both diabetic constructs relative to the healthy (Fig. 3C, D). In contrast, the number of metabolites uniquely altered between the healthy and T1DM constructs was 21, with the majority upregulated in the T1DM (Fig. 3C, E). The number of metabolites between the healthy and T2DM was 22; interestingly, the majority were downregulated in the T2DM (Fig. 3C, F).

### T2DMs exhibit decreased capacity for glucose metabolism relative to T1DM

**Figure 4:**
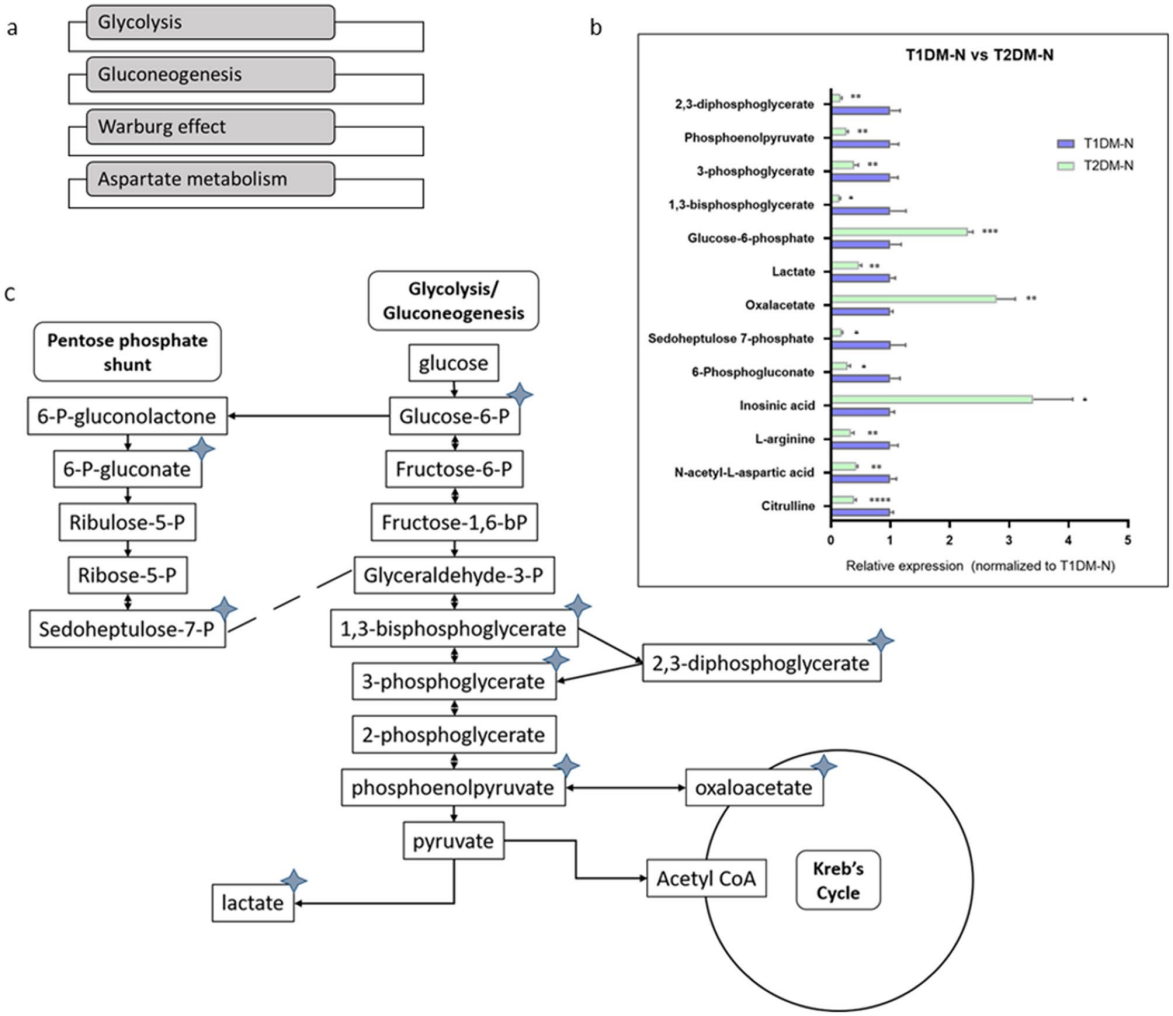
(**A**) The top four pathways altered between T1DM and T2DM innervated constructs. (**B**) graph of the relative concentration of metabolites found in the top 4 most significant pathways of T2DM and T1DM innervated constructs. (**C**) Representative diagram of significant metabolites (indicated in blue stars) on the main pathways involved in glucose metabolism. n = 3–4, student’s t test, p: *≤ 0.05; **≤ 0.01; ***≤ 0.001; ****≤ 0.0001.

In order to further elucidate key distinctions between the metabolic profiles of T1DM and T2DM corneal pathophysiology, we conducted enrichment and pathway analyses between the innervated T1DM and T2DM constructs. In total, 61 metabolites were found to be significantly altered between the two disease types, with 83% of them at lower levels in the T2DM constructs than the T1DM (Fig. 4A). The top four pathways that these metabolites fell under were generally associated with glucose metabolism, including gluconeogenesis (p = 0.00251), glycolysis (p = 0.0109), the Warburg effect (p = 0.0133), as well as aspartate metabolism (p = 0.0144) (Fig. 4A). Interestingly, the T2DM cultures exhibited decreased levels of metabolites associated with glucose metabolism, with the exception of glucose-6-phosphate and oxaloacetate, which were found to be higher in the T2DM than the T1DM (Fig. 4B). The diagram in Fig. 4C indicates the position of the contributing metabolites in relevant pathways, including gluconeogenesis, glycolysis, the Krebs cycle and the pentose phosphate pathway.

## Discussion

DM is a multifactorial systemic disease that remains difficult to manage by therapeutic intervention due to a complex and elusive pathophysiology that has resulted from prolonged elevated blood glucose levels^20^. DM can generally be characterized by a reduction in insulin signaling at the cellular level, causing dysfunctional glucose metabolism^21^. Cellular metabolism is highly sensitive to changes and its coordination is dependent upon a well-balanced system, so a dysfunctional glucose metabolism can disrupt numerous metabolic pathways, including lipid and amino acid metabolism^22,23^. These changes, when occurred, can lead to large scale systemic pathologies including hyperglycemia and hyperlipidemia, which only perpetuates a cyclical pathogenesis and further exacerbates the symptoms. Moreover, these metabolic pathways continue to be implicated in the development and onset of T2DM, and they are considered to contribute strongly to the many different systemic pathologies that occur, including diabetic retinopathy, nephropathy, and keratopathy^6,24,25^.

Diabetic keratopathy is a common secondary pathology of DM and presented clinically as spontaneous corneal lesions, stromal edema and clouding, epithelial fragility, neuronal degeneration, and reduced/slow wound healing, with patients having a higher risk of post-operative complications^9^. A strong suspect to be causing these complications is reduced corneal innervation, which has been heavily documented^13^. One of the stromal abnormalities that occurs in the diabetic cornea is stiffening, believed to be a result of fibrotic ECM secretion^17^. There is a relationship between neuron and stromal fibroblast activity^15^, and as a disease of metabolic nature, we investigated how our co-culture of these cell types can affect metabolic activity, and how this could vary in diabetic cells. Metabolic reprogramming is a process that occurs upon fibroblast activation^26^, and these abnormalities in ECM secretion have been linked to mitochondrial dysfunction^27^.

Our 3D self-secreted ECM innervated corneal model utilizes stromal fibroblasts from human diabetic donors, onto which we culture differentiated sensory neurons from the SH-SY5Y cell line to replicate the native innervated corneal stroma. With this model, we have been able to study how the neurons relate to the metabolic function of the diabetic stroma, and how the diabetic stroma differs metabolically from a healthy stroma. Specifically, we have utilized this model to elucidate key metabolic differences of T1DMs and T2DMs, from HCFs, by identifying differentially expressed metabolites and the metabolic pathways in which these are produced. To our knowledge, we are the first group to utilize metabolomics to investigate the diabetic corneal stroma and dissect the interplay/interactions with the neurons. Metabolomics has become a more common application in diabetic studies^28–30^, often for the identification of early metabolic alterations that occur during the development of T2DM; however, here we have used the analytic technique in the context of diabetic keratopathy. Our data shows that the addition of neurons to diabetic stroma ECMs resulted in a markedly different shift in metabolism than when added to healthy stroma ECM. Specifically, the addition of neurons to the healthy constructs supported basic energy production pathways, including glycolysis, the glucose phosphate shuttle, and the electron transport chain. On the other hand, neuron addition to the diabetic constructs upregulated metabolites associated with amino acid and nucleic acid metabolic pathways, including methionine, aspartate, and purine metabolism. Purines are utilized in many different biochemical processes, including cellular metabolism and signal transduction^31^, and can therefore impact many different functions of the cell. The metabolites contributing to the observed change in purine metabolism are found to be both up- and down-regulated (see Supplemental Table 1), indicating that a more complex interplay is occurring within the cell, other than a simple up- or down-regulation. A metabolite of note is myoinositol, which was upregulated in both the T1DM and T2DM constructs. This metabolite was also found to be upregulated by neuron addition in both T1DMs and T2DMs, but not the healthy constructs. Myoinositol has been implicated in the promotion of insulin signaling^32^, and has been found at elevated levels in diabetic urine^33^. Our data could support that myoinositol dysregulation in DM is related to the neuronal dysfunction that occurs in diabetic keratopathy. Furthermore, these data suggest that neuron interaction within the diabetic stroma is defined by a markedly different profile from the healthy stroma-neuronal crosstalk, in the context of biosynthetic metabolic pathways.

Dysfunction in amino acid metabolism has been documented in T2DM^23,24^ and abnormal levels of both branch chain and aromatic amino acids have been found in the plasma of T1DM and T2DM patients^30^. Since our study focuses on cell-cell interactions and metabolite modulation within the tissues rather than those found in circulation, this could account for possible differences in the types of amino acids found. Interestingly, while the addition of neurons activated similar pathways in both T1DMs and T2DMs, a comparison of the innervated diabetic constructs to the healthy innervated constructs showed that several pathways are unique for T1DMs and T2DMs. While the T1DM innervated constructs exhibited an upregulation in biosynthetic amino acid and phospholipid pathways compared to the healthy, the T2DMs exhibited decreased levels of amino acids arginine and glutathione (Fig. 3D).

Oxidative stress is known to contribute to the T2DM pathophysiology^34^, and arginine and glutathione have been found to reduce oxidative stress in an experimental model^35^. Therefore, our results may suggest that the T2DM constructs experience increased levels of oxidative stress as a result of decreased levels of arginine and glutathione. T1DMs showed upregulated metabolites normally found in more biosynthetic pathways, including those for phospholipids and amino acids, while the most highly differentially expressed metabolites between the T2DM and healthy are those of energy-producing pathways. Specifically, the majority of these metabolites were downregulated, suggesting that T2DMs have a less sufficient ATP production system than HCFs. Despite the differences found between T1DMs and T2DMs, they did exhibit some metabolites that were expressed in similar patterns, supporting that there is some commonality among their metabolic profiles. We found 9 of the 13 significantly modulated metabolites between the innervated T1DMs and T2DMs were found in main ATP producing pathways, mostly related to the glycolysis pathway. Furthermore, most of these metabolites were downregulated in T2DMs, with the exception of glucose-6-phosphate and oxaloacetate. This suggests that while both diseases possess a similar dysfunction in their capacity for glucose metabolism, as exhibited by their lack of proper insulin signaling, the specific biochemical processes within the cell related to glucose metabolism differ between the two disease states.

DM is a disease of a dysfunctional metabolism, and metabolomics can provide great insight into the specific biomolecular events that occur within the cells and tissues as a result of the disease. Our study utilizes metabolomics to delineate the impact of corneal innervation on the metabolic function of the diabetic corneal stroma, as well as to understand differences in the metabolism between T1DMs and T2DMs. In fact, the differences between T1DM and T2DM in the context of the corneal pathobiology are often ignored or not examined. Our in vitro 3D co-culture model comprised of native corneal stromal cells and SH-SY5Y sensory neurons allows for detection of molecular changes, and can provide critical insights into long-lasting metabolic defects as a result of DM. Consequently, this could also mask metabolic alterations that occur in real-time due to the in vivo diabetic environment, and due to these limitations of the current model, studies conducted within a hyperglycemic and hyperlipidemic in vitro setting are warranted. Our studies herein provide a first insight into the relationship between corneal innervation and the metabolic pathophysiology of the diabetic corneal stroma and provide the basis for future metabolic studies within the cornea.

## Supplementary information

Supplementary Table 1.

Supplementary Table 2.
