# Supplementary material for: Nerve influence on the metabolism of type I and type II diabetic corneal stroma: an in vitro study
Source: Sci Rep. 2021 Jul 1;11:13627. doi: 10.1038/s41598-021-93164-1 (PMC8249404; doi:10.1038/s41598-021-93164-1)
Supplement: Supplementary file 1 — Supplementary Table 1. [file 41598_2021_93164_MOESM1_ESM.pdf]

Nerve influence on the metabolism of type I and type II diabetic corneal stroma: An in vitro study

Amy E. Whelchel[1], Sarah E. Nicholas[2,3], Jian-Xing Ma[1,4], Dimitrios Karamichos[2,3,5*]

comparison	top 4 pathways	p-value	contributing metabolite	p-value	regulation
healthy/ healthy-N	glycerol phosphate shuttle	0.00768	*glyceric acid 1,3-biphosphate*	*0.01265*	*down*
	electron transport chain	0.0225	2,3-diphosphoglyceric acid	0.00716	down
	**pyrimidine metabolism**	0.0344	**dTDP**	**0.01808**	**up**
	glycolysis	0.0379	**Thymidine 5'-triphosphate**	**0.00447**	**up**
			glycerol 3-phosphate	*0.02806*	*up*
			**thymidine**	**0.02125**	**up**
T1DM/ T1DM-N	pyrimidine metabolism	0.00975	*Adenosine*	*0.01778*	*down*
	purine metabolism	0.0247	***Adenosine monophosphate***	***0.01011***	***down***
	**aspartate metabolism**	0.0453	L-Cystathionine	0.00130	up
	methionine metabolism	0.0755	*Deoxyadenosine triphosphate*	*0.00101*	*up*
			dCMP	0.00030	up
			dCTP	0.01170	up
			Deoxyguanosine	*0.00170*	*up*
			Thymidine 5'-triphosphate	0.00043	up
			Deoxyuridine triphosphate	0.00798	up
			Inosinic acid	***0.03444***	***down***
			**N-Acetyl-L-aspartic acid**	**0.00270**	**up**
			Thymidine	0.00859	up
T2DM/ T2DM-N	purine metabolism	0.00024	*Phosphoribosyl pyrophosphate*	*0.02058*	*up*
	pyrimidine metabolism	0.000266	**Adenosine**	0.01278	down
	**methionine metabolism**	0.00154	**Adenosine monophosphate**	**0.03470**	**down**
	aspartate metabolism	0.0036	**Choline**	**0.00023**	**up**
			**L-Cystathionine**	**0.00005**	**up**
			Cytidine	*0.01009*	*down*
			Deoxyadenosine triphosphate	0.00455	up
			dCDP	*0.01588*	*up*
			dCMP	*0.00014*	*up*
			dCTP	*0.00051*	*up*
			Deoxyguanosine	0.00498	up
			Thymidine 5'-triphosphate	*0.00339*	*up*
			Guanine	0.00190	down
			Guanosine	0.00101	down
			Hypoxanthine	0.02317	down
			Argininosuccinic acid	0.00020	up
			N-Acetyl-L-aspartic acid	0.00064	down
			Ureidosuccinic acid	*0.00933*	*up*
			**Sarcosine**	**0.00032**	**up**

			Pyrophosphate	*0.01766*	*up*
healthy-N/ T1DM-N	methionine metabolism	0.0025	**L-serine**	**0.00002**	**up**
	aspartate metabolism	0.00538	homocysteine	0.00165	up
	**phosphatidylethanolamine biosynthesis**	0.00652	5'-methylthioadenosine	0.04852	up
	pyrimidine metabolism	0.0124	5-methyltetrahydrofolic acid	0.01386	up
			putrescine	0.03175	down
			methionine sulfoxide	0.00671	up
			fumaric acid	*0.00005*	*up*
			L-asparagine	*0.00064*	*up*
			L-aspartic acid	*0.00026*	*up*
			N-acetyl-L-aspartic acid	*0.01045*	*up*
			ureidosuccinic acid	*0.00649*	*up*
			**cytidine triphosphate**	**0.00005**	**up**
			**CDP-ethanolamine**	**0.00089**	**up**
			cytidine	0.00334	up
			phosphoribosyl pyrophosphate	0.03849	up
			dCDP	0.04116	up
			thymidine 5'-triphosphate	0.00021	up
healthy-N/ T2DM-N	electron transport chain	0.00308	*Glyceric acid 1,3-biphosphate*	*0.00557*	*up*
	glycerol phosphate shuttle	0.00501	2,3-Diphosphoglyceric acid	0.02612	up
	**aspartate metabolism**	0.00538	**L-Arginine**	**0.04342**	**down**
	gluconeogenesis	0.00538	**L-Aspartic acid**	**0.00084**	**up**
			Dihydroxyacetone phosphate	*0.00584*	*down*
			**Fumaric acid**	**0.00006**	**up**
			L-Lactic acid	0.03291	down
			**Ureidosuccinic acid**	**0.01063**	**up**
			**Oxalacetic acid**	**0.00850**	**up**
			Glycerol 3-phosphate	*0.02108*	*down*
T1DN-N/ T2DM-N	gluconeogenesis	0.00251	**L-lactic acid**	**0.00368**	**down**
	glycolysis	0.0109	oxalacetic acid	0.00908	up
	**Warburg effect**	0.0133	***phosphoenolpyruvic acid***	***0.01142***	***down***
	aspartate metabolism	0.0144	***3-phosphoglyceric acid***	***0.00957***	***down***
			glyceric acid 1,3-biphosphate	***0.04774***	***down***
			2,3-diphosphoglyceric acid	***0.01397***	***down***
			glucose-6-phosphate	***0.00252***	***up***
			inosinic acid	0.03528	up
			L-arginine	0.00854	down
			N-acetyl-L-aspartic acid	0.00967	down
			citrulline	0.00010	down
			**D-sedoheptulose 7-phosphate**	**0.04683**	**down**
			**6-Phosphogluconic acid**	**0.04558**	**down**

Supplemental Table 1.) Metabolic impact of neuronal presence in healthy and diabetic constructs. Detailed list of contributing metabolites that determined the top pathways involved via Metaboanalyst.
