# Supplementary material for: Nerve influence on the metabolism of type I and type II diabetic corneal stroma: an in vitro study
Source: Sci Rep. 2021 Jul 1;11:13627. doi: 10.1038/s41598-021-93164-1 (PMC8249404; doi:10.1038/s41598-021-93164-1)
Supplement: Supplementary file 2 — Supplementary Table 2. [file 41598_2021_93164_MOESM2_ESM.pdf]

Nerve influence on the metabolism of type I and type II diabetic corneal stroma: An in vitro study

Amy E. Whelchel[1], Sarah E. Nicholas[2,3], Jian-Xing Ma[1,4], Dimitrios Karamichos[2,3,5]*

Metabolite	p-value	FDR
aspartate	7.3400E-08	1.8800E-05
2-keto-isovalerate	4.4900E-07	5.7400E-05
p-hydroxybenzoate	9.8900E-07	8.4400E-05
fumarate	2.2000E-06	1.4000E-04
shikimate	5.5400E-06	2.8000E-04
pantothenate	9.3300E-06	3.5000E-04
Maleic acid	9.5400E-06	3.5000E-04
2,3-dihydroxybenzoic acid	1.3100E-05	4.2000E-04
oxaloacetate	1.7400E-05	5.0000E-04
serine	2.6300E-05	6.7000E-04
proline	3.0300E-05	6.7000E-04
histidine	3.4500E-05	6.7000E-04
deoxyguanosine	3.5700E-05	6.7000E-04
Guanidoacetic acid	3.7600E-05	6.7000E-04
1-Methyl-Histidine	3.9300E-05	6.7000E-04
dATP-nega	4.6900E-05	7.5000E-04
dTTP-nega	5.3500E-05	8.1000E-04
myo-inositol	5.9000E-05	8.4000E-04
cystathionine	7.2000E-05	9.7000E-04
CTP-nega	8.3000E-05	1.0600E-03
malate	1.1000E-04	1.3500E-03
1,3-diphopshateglycerate	1.2000E-04	1.3500E-03
choline	1.3000E-04	1.4000E-03
allantoate	1.5000E-04	1.5700E-03
4-phosphopantothenate	1.7000E-04	1.6300E-03
glutamate	1.7000E-04	1.6300E-03
D_nega	1.7000E-04	1.6300E-03
dihydroxy-acetone-phosphate	1.8000E-04	1.6300E-03
CDP-ethanolamine	1.8000E-04	1.6300E-03
2,3-Diphosphoglyceric acid	2.1000E-04	1.7600E-03
leucine-isoleucine	3.1000E-04	2.5600E-03
Phenyllactic acid	3.2000E-04	2.5600E-03
glyoxylate	3.3000E-04	2.5600E-03
valine	3.6000E-04	2.7200E-03
D-sedoheptulose-1-7-phosphate	4.6000E-04	3.3000E-03
adenine	4.9000E-04	3.3000E-03

Methylcysteine	4.9000E-04	3.3000E-03
asparagine	4.9000E-04	3.3000E-03
dehydroascorbic acid	5.3000E-04	3.4700E-03
S-adenosyl-L-homoCysteine-posi	5.9000E-04	3.7600E-03
DP_posi	6.2000E-04	3.8900E-03
lactate	7.1000E-04	4.3300E-03
tyrosine	7.5000E-04	4.4800E-03
homocysteine	8.6000E-04	4.9400E-03
xanthosine	8.7000E-04	4.9400E-03
homocysteic acid	9.5000E-04	5.2200E-03
phosphocreatine	9.6000E-04	5.2200E-03
DL-Pipecolic acid	9.8000E-04	5.2200E-03
citrate	1.0300E-03	5.2700E-03
N-carbamoyl-L-aspartate-nega	1.0300E-03	5.2700E-03
sn-glycerol-3-phosphate	1.0500E-03	5.2700E-03
5-phosphoribosyl-1-pyrophosphate	1.1800E-03	5.8000E-03
guanine	1.2200E-03	5.8900E-03
phosphoenolpyruvate	1.2600E-03	6.0000E-03
3-hydroxybuterate	1.3400E-03	6.2200E-03
Kynurenine	1.3600E-03	6.2200E-03
arginine	1.5000E-03	6.7300E-03
guanosine	1.6100E-03	7.0900E-03
citrulline	1.7100E-03	7.4200E-03
S-adenosyl-L-methionine	1.7400E-03	7.4200E-03
glutamine	1.8200E-03	7.6400E-03
Adenylosuccinate	1.8500E-03	7.6400E-03
cytidine	1.9200E-03	7.8200E-03
Ng,NG-dimethyl-L-arginine	1.9700E-03	7.8600E-03
2-hydroxygluterate	2.1100E-03	8.3200E-03
nicotinamide	2.2000E-03	8.4400E-03
inosine	2.2100E-03	8.4400E-03
D_posi	2.3400E-03	8.8300E-03
hexose-phosphate	2.5000E-03	8.9300E-03
N-acetyl-glucosamine-1-phosphate	2.5100E-03	8.9300E-03
creatine	2.5100E-03	8.9300E-03
allantoin	2.5100E-03	8.9300E-03
Phosphorylcholine	2.7400E-03	9.6200E-03
lysine	2.8300E-03	9.7400E-03
methionine	2.8500E-03	9.7400E-03
Phenylpropiolic acid	2.9400E-03	9.9100E-03
phenylalanine	2.9900E-03	9.9400E-03
a-ketoglutarate	3.2400E-03	1.0640E-02
dGTP	3.3400E-03	1.0830E-02

L-arginino-succinate	3.4700E-03	1.1100E-02
2-ketohaxanoic acid	4.1500E-03	1.3110E-02
Glycerophosphocholine	4.2500E-03	1.3280E-02
arginosuccinic acid	4.5300E-03	1.3960E-02
FMN	4.8700E-03	1.4830E-02
indole	4.9600E-03	1.4930E-02
UDP-D-glucuronate	5.1600E-03	1.5360E-02
Creatinine	5.2800E-03	1.5550E-02
betaine	5.6600E-03	1.6460E-02
uridine	5.9400E-03	1.7090E-02
purine	6.3300E-03	1.7690E-02
CMP	6.3400E-03	1.7690E-02
4-aminobutyrate	6.3600E-03	1.7690E-02
2-dehydro-D-gluconate	6.5200E-03	1.7770E-02
threonine	6.5200E-03	1.7770E-02
Uric acid	6.8200E-03	1.8390E-02
dimethylglycine	7.2800E-03	1.9410E-02
putrescine	7.8900E-03	2.0820E-02
cytosine	8.3700E-03	2.1770E-02
tryptophan	8.4200E-03	2.1770E-02
cholesterol	8.5500E-03	2.1900E-02
7-methylguanosine	8.8300E-03	2.2380E-02
Aminoadipic acid	8.9900E-03	2.2550E-02
Indole-3-carboxylic acid	9.1400E-03	2.2710E-02
UTP-nega	9.3400E-03	2.2990E-02
CDP-nega	9.6400E-03	2.3360E-02
Xanthurenic acid	9.6700E-03	2.3360E-02
N-acetyl-L-aspartylglutamic acid	9.9900E-03	2.3900E-02
Atrolactic acid	1.0950E-02	2.5950E-02
N-Acetyl-L-alanine	1.1330E-02	2.6520E-02
dCTP-nega	1.1400E-02	2.6520E-02
alanine	1.1690E-02	2.6960E-02
N-acetyl-L-aspartic acid	1.1820E-02	2.7010E-02
Carbamoyl phosphate	1.2790E-02	2.8970E-02
3-phosphoglycerate	1.3060E-02	2.9320E-02
CDP-choline	1.4020E-02	3.1170E-02
Methionine sulfoxide	1.4130E-02	3.1170E-02
thiamine-phosphate	1.7210E-02	3.7650E-02
ATP-nega	1.8200E-02	3.9490E-02
thymine	1.8560E-02	3.9920E-02
Kynurenic acid	1.9450E-02	4.1380E-02
N-acetyl spermidine	1.9560E-02	4.1380E-02
S-ribosyl-L-homocysteine-posi	2.0820E-02	4.3690E-02

trehalose-6-Phosphate	2.2610E-02	4.7070E-02
acetyl-CoA-posi	2.3160E-02	4.7810E-02
dCDP-nega	2.3540E-02	4.8200E-02

Supplemental Table 2.) T1DMs and T2DMs exhibit significant alternations in key energy production metabolic processes. Detailed list of metabolic differences analyzed between healthy and diabetics, representing 125 metabolites significantly altered between healthy and diabetic innervated cultures. Analysis was conducted by a one-way ANOVA, and post-doc was completed through Fisher's LSD Method.
